# Opiate System Mediate the Antinociceptive Effects of *Coriandrum sativum* in Mice

**Published:** 2012

**Authors:** Abbas Ali Taherian, Abbas Ali Vafaei, Javad Ameri

**Affiliations:** a *Laboratory of Pain, Research Center and Department of Physiology, Semnan University of Medical Sciences, Semnan, Iran. *; b *Research Center of Medicinal Plant, Semnan University of Medical Sciences, Semnan, Iran.*

**Keywords:** Coriandrum sativum, Pain, Dexamethasone, Stress, Opiate system, Mice

## Abstract

Our previous study showed that *Coriandrum sativum *(CS) has antinociceptive effects, but the mechanisms that mediate this effect are not clear. The present study was designed to test the role of opiate system in the antinociceptive effects of CS on acute and chronic pain in mice using Hot Plate (HP), Tail Flick (TF) and Formalin (FT) tests and also to compare its effect with dexamethasone (DEX) and stress (ST).

Young adult male albino mice (25-30 g) in 33 groups (n = 8 in each group) were used in this study. CS (125 250, 500 and 1000 mg/Kg IP), DEX (0.5, 1 and 2 mg/Kg IP), vehicle (VEH) or swim stress were used 30 min before the pain evaluation tests. Acute and chronic pain was assessed by HP, TF and FT models. In addition, Naloxone (NAL, 2 mg/Kg, IP) was injected 15 min before the CS extract administration in order to assess the role of opiate system in the antinociception of CS.

Results indicated that CS, DEX and ST have analgesic effects (p < 0.01) in comparison with the control group and higher dose of CS was more effective (p < 0.001). Besides, pretreatment of NAL modulates the antinociceptive effects of CS in all models (p < 0.001).

The above findings showed that CS, DEX and ST have modulator effects on pain. These findings further indicate that the CS extract has more analgesic effects than DEX and ST and also provides the evidence for the existence of an interaction between antinociceptive effects of CS and opiate system.

## Introduction

Coriander is native to the Mediterranean and Middle Eastern region. The etymology of coriander starts with the Greek word korannon, a combination of koris and annon (a fragrant anise) and referred to the ripe fruit (1). The seeds (fruits) of *Coriandrum sativum* L (CS) are widely used to season curries, puddings, breads, sausages, liqueurs, cakes, gin essences and spicy sauces (2).

The CS is a grass-like plant belonging to the Apiaceae family. This plant in Iranian traditional medicine is believed to have carminative, diuretic, and affect in dyspeptic complaints, loss of appetite, convulsion, insomnia and anxiety and in medical purposes, the fruits of coriander have been used in different forms (powdered seeds or dry extract) (3, 4). It has been reported that CS has antihypertensive (5), antifertility (6), antihyperglycemic (7-11), antihyperlipidemic (12, 13), antiproliferative (14), antioxidant (15, 16), anticonvulsant (17), antinociceptive (18, 19), and anxiolytic effects (20, 21) which decrease the toxic heavy metal accumulation in fish (22).

On the other hand, our previous studies showed that Glucocorticoids and stress have analgesic effects (23, 24) and one of the mechanisms involved in these effects was opioidergic system (25). Besides, some previous studies suggested that there is probably an interaction between the antinociceptive effects of CS and the opioidergic system.

To our knowledge, no studies have been performed on the role of opiate receptors on the antinociceptive effect of CS and in comparison with dexamethasone (DEX) and stress (ST) so far. The present study was designed to examine the analgesic effects of the aqueous extract of CS seeds on acute, chronic (neurogenic and inflammatory pain) in HP, TF and FT and compare its effects with DEX and ST in mice. The HP and TF tests measured the response to brief noxious stimuli. The animal’s responses in these tests were integrated at rather low levels in the central nervous system, probably giving information mainly about the pain threshold. On the other hand, FT measured the response to a long-lasting nociceptive stimulus and thus may have a closer resemblance to clinical pain. In FT test, two types of pain were postulated: a short lasting pain caused by a direct effect on nociceptors followed by a long lasting pain due to the inflammation. Furthermore, in this research we used Naloxone (a non-selective opiate antagonist) to determine the participation of the opiate system in the antinociceptive effect of CS extract.

## Experimental

*Animals*

Young adult male albino mice (each 25-30 g) obtained from the animal house of physiological research center of Semnan University of medical sciences (Semnan, Iran) were used in this study. They were housed in 7 standard clear plastic cages (n = 7) at 22 ± 2^o ^C in 12 h light/ 12 h dark cycle (6 AM lights on), with free access to food and water. All the investigations were conducted in the light phase on a natural light cycle, between 9.00 and 14.00 and the animals were used only once in each test. All procedures were conducted in agreement with the National Institutes of Health Guide for care and use of laboratory animals.

*Plant material*

**Figure 1:**
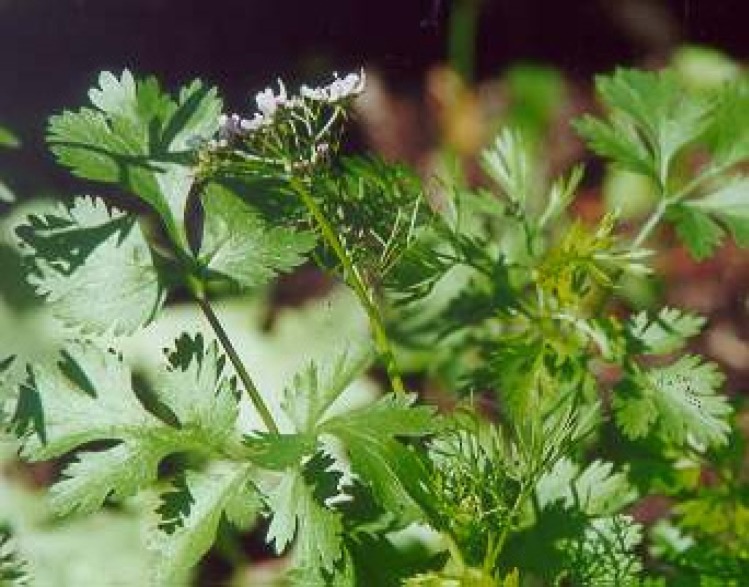
Coriandrum sativum.

**Figure 2:**
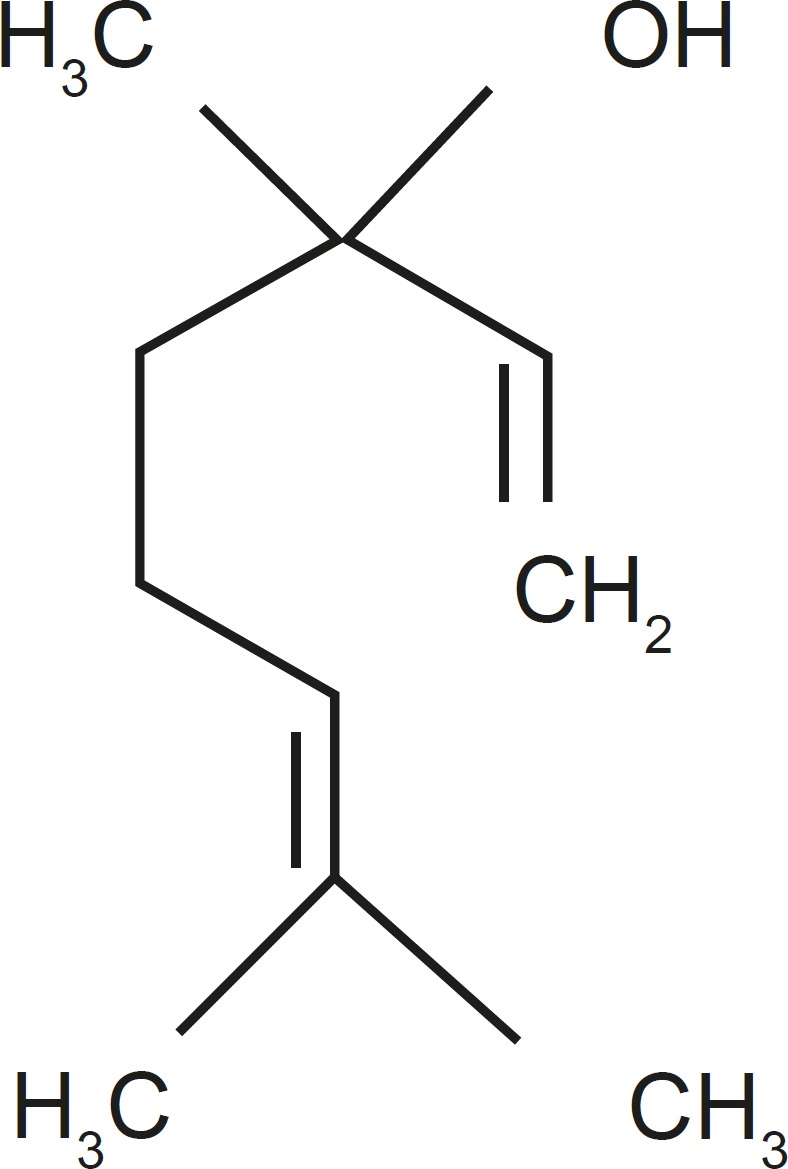
Chemical structure of Linalool as an important constituent of Coriander oil.

The dried seeds of CS plant were collected from Semnan Botanicals Garden (Semnan, Iran). The plant was identified by Dr. Etemadi in the Center of Academic and Practical of Agricultural Sciences located in Semnan (Semnan, Iran) and the voucher specimen (No. 83-32) was preserved and deposited in the herbal library of the mentioned center. For preparing the extract, 100 g of the seeds from the air-dried material of CS were exhaustively extracted with 1000 mL water by soxhlet extraction (26). The extract of CS (125, 250, 500 and 1000 mg/Kg) or vehicle were injected intraperitoneally 30 min before the pain assessment tests. The drugs’ doses were mainly derived from the pilot studies and another research in medicinal plants (27, 28). In addition, previous studies indicated that the main component of CS is Linalool (Figures 1 and 2).

*Dexamethasone*

Dexamethasone (DEX, Synopharm, Italy) as a specific GR agonist (0.5, 1 and 2 mg/Kg) or vehicle (VEL) was injected intraperitoneally in the same time of CS injection. DEX was dissolved initially in 100% ethanol and diluted to a final concentration of 2% ethanol in 0.9% saline. A 2% ethanol solution in saline was used for vehicle control injection.

*Naloxone*

Naloxone (NAL) 2 mg/Kg IP (Sigma Co.) as a non-selective opioid receptors antagonist was injected into animals to investigate the participation of opiate system in the antinociceptive effect of the CS extracts 15 min before the administration of the best dose of CS extract (1000 mg/Kg) according to the method indicated in previous study (29). NAL was initially dissolved in SAL. Drugs and solutions were freshly prepared before each experiment.

*Acute toxicity (Lethal does)*

After the injection of the extract into each group, the number of possible mortality cases and behavioral changes was counted during a 72 h period (30).

*Swim Stress*

For inducing the stress, animal was made to swim for 1 min in cold water (18-22°C).

*Pain assessment tests and apparatuses*

*Tail flick*

The tail flick test is a standard model for evaluating the nociception in animals. In this test, we used the tail-flick apparatus which was described by D›Amour and Smith in 1941. In this research, we used TF task (Ugo Basile Co., Spain) to measure the acute nociceptive responses (31). The animal’s tail was placed over a window located on the apparatus platform. A switch activated a light beam to heat the animal tail with certain intensity that was selected via a knob. The Light beam intensity was scaled from 0 to 10. Scale 5 was used for this experiment. When the animal felt discomfort, it flicked its tail which automatically stopped the light beam and timer. The reaction time was automatically presented on a digital display. The latency of the mouse to remove its tail from the heat is recorded. In our studies, the maximum time of heat exposure (“cut-off” time) to avoid tissue damage was 13 sec (32).

*Hot plate*

This test is a model of supraspinal analgesia (33). The hot plate test also uses heat as the noxious stimulus. The method was used by Wolf and McDonald in 1994. In this research, we used the HP task that was produced by Harvard Co. (Harvard Co., USA). In this model, the animal is placed on a hot plate and the response time is measured (34). The hot plate setup used in this experiment is consisted of a plate (diameter = 19 cm) and an open-ended Plexiglas cylinder (height = 25 cm)**,** located around the plate to prevent the animal from escaping. The plate was heated by a thermode and its temperature was fixed using a thermostat in 52.5 ± 0/5°C. The time it takes for the animal to respond to the stimuli, by either licking its paw or by jumping up from the plate, was measured as reaction time. To avoid tissue damage, “cut-off” time was set at 30 sec (35, 36).

*Formalin test*

Unlike the hot plate and tail flick tests, the formalin test uses formalin solution as a chemical noxious stimulus. The formalin test is an important animal model in the study of acute long-lasting pain (37). The method used in this study was determined using the formalin test as described by Dubuission and Dennis (38).

Intraplantar injection of formalin solution causes the animal to lick its injected paw. This behavior is seen in three phases: the initial phase that occurs between 0 and 5 min after the injection and produces due to the direct stimulation of nociceptors (neurogenic pain); the quiescent phase during which the animal does not show licking behavior (between 5 and 15 min after the injection) and the third phase that occurs between 15^th^ and 60^th^ min due to the inflammatory process (inflammatory pain) (32, 38, 39). The observation setup consisted of an 4-legged aluminum with a plate of glass located above it. Animal was placed on this glass plate below an open-ended Plexiglas cylinder. To observe animal’s licking behavior, a mirror was located below the glass plate. To be adapted to the apparatus and the environment, animals were placed on the glass plate for 30 min, 2 days before the initiation of the experiments and before the formalin injection on the experiment day. On the test day, 25 µL of 3% formalin (Merck, Germany) was injected intradermally into the right hind paw of each animal. Then, the time of licking or climbing behaviors were measured for 60 min.

**Figure 3:**
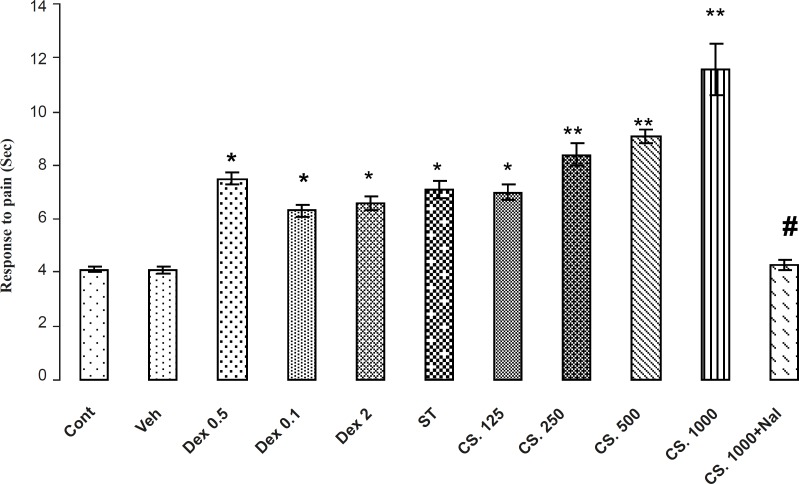
Effects of aqueous extract of CS seeds, DEX and ST groups on the pain responses in Tail Flick model. The administration of the CS extract (125, 250, 500 and 1000 mg/Kg IP) or DEX (0.5, 1 and 2 mg/Kg IP) or ST group 30 min before the Tail Flick test increased the reaction time. In addition, the pretreatment NAL reversed this effect. Data are expressed as the mean ± SEM. *p < 0.01, **p < 0.001 as compared with Cont or VEH groups, # p < 0.01 as compared with CS 1000.

*Statistics*

Data were analyzed by one-way and two-way analysis of variance (ANOVA), followed by Tukey’s test for multiple comparisons. Values of p < 0.05 were considered as significant.

*Experiment 1*

The aim of this experiment was to determine the effects of systemic injection of CS seeds’ extract on pain sensation in tail flick model. Mice were randomly divided into eleven groups (n = 7 in each group) as follows: Control group: No injection (n = 7). Vehicle group: At the same volume of the extract (n = 7). CS extract groups: Including 4 groups (n = 28). DEX groups: Including 3 groups (n = 21). ST group: All injections were done 30 min before Tail Flick test (n = 7). NAL group: Injected 15 min before the best dose (1000 mg/Kg) of CS extract injection (n = 7)

*Experiment 2*

In this experiment, the effects of systemic injection of CS seeds’ extract on pain sensation were examined in hot plate model. Mice were randomly divided into eleven groups (n = 7 in each group) as follows: Control group: No injection (n = 7). Vehicle group: At the same volume of the extract (n = 7). CS extract group: Including 4 groups (n = 28). DEX group: Including 3 groups (n = 21). ST group: All injections were done 30 min before the Hot Plate test (n = 7). NAL group: Injected 15 min before the best dose (1000 mg/Kg) of CS extract injection (n = 7)

**Figure 4:**
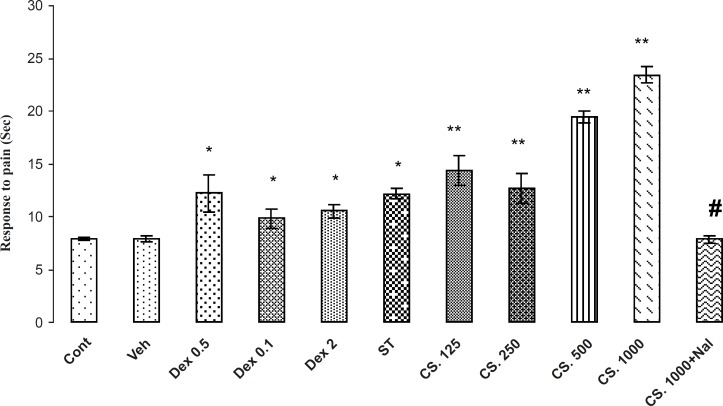
Effects of the aqueous extract of CS seeds, DEX and ST on pain responses in Hot plate model. The administration of the CS extract (125, 250, 500 and 1000 mg/Kg IP) or DEX (0.5, 1 and 2 mg/Kg IP) or ST 30 min before the Hot plate test increased the reaction time (p < 0.01). Besides, the pretreatment of NAL reversed this effect. Data are expressed as the mean ± SEM. *p < 0.01, **p < 0.001 as compared with Cont or VEH groups,# p < 0.01 as compared with CS 1000.

*Experiment 3*

The aim of the third experiment was to determine the effects of systemic injection of CS seeds extract on neurogenic and inflammatory pain. Mice were randomly divided into eleven groups (n = 7 in each group) as follows: Control group: No injection (n = 7). Vehicle group: At the same volume of the extract (n = 7). CS extract group: Including 4 groups (n = 28). DEX group: Including 3 groups (n = 21). ST group: All injections were done 30 min before the Formalin test (n = 7). NAL group: Injected 15 min before the best dose (1000 mg/kg) of CS extract injection (n = 7)

## Results and Discussion

*Acute toxicity*

CS extract (125-1000 mg/Kg IP) had no mortality during the observation period 72 h after the administration.

*Tail flick*

The results of antinociceptive effects of CS extract, DEX and ST on Tail flick are demonstrated in Figure 3. The Analysis of the data in the control and treatments groups indicated that CS extract, DEX and ST, significantly (p < 0.01) increased the reaction time in a dose-dependent manner so that the extract-treated mice showed an increased reaction time in comparison with the vehicle-treated and control animals in tail flick test. In addition, animals that received 1000 mg/Kg of CS extract had a higher reaction time in comparison to the other groups. Pretreatment of NAL (2 mg/Kg) at the best dose of the CS extract (1000 mg/Kg) attenuated the reaction time in tail flick test. Thus, NAL attenuates antinociception effects of CS extract.

*Hot plate*

The results of the antinociceptive effects of CS extract, DEX and ST on the Hot Plate are demonstrated in Figure 4. The analysis of the data indicated that the CS extract, DEX and ST have significantly antinociceptive effects in comparison to the VEH or control groups (p < 0.01). In addition, animals that received 1000 mg/Kg of CS extract, have the most reaction time in this test and NAL (2 mg/Kg) at this dose, attenuated the reaction time in tail flick test. Thus, NAL attenuates antinociception effects of the CS extract.

*Formalin test*

**Figure 5:**
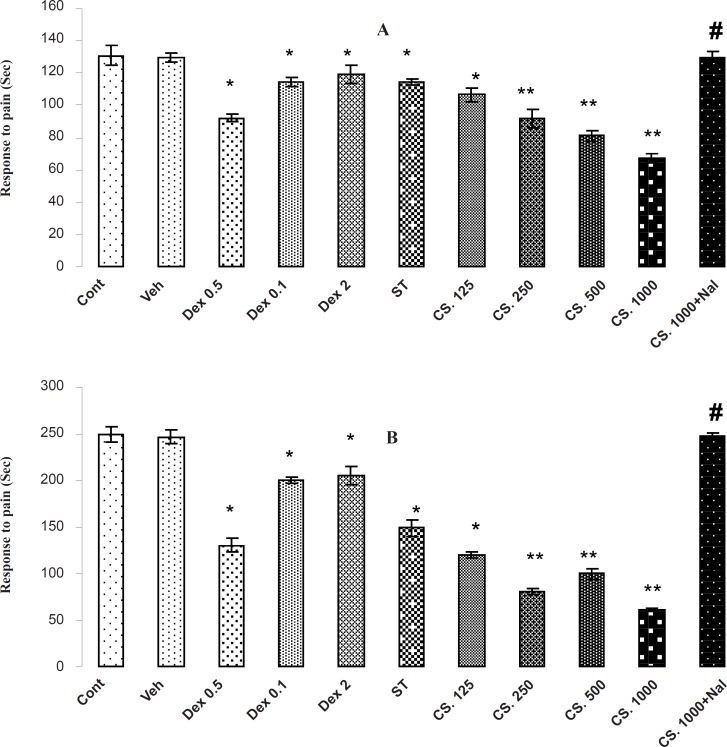
Effects of the aqueous extract of CS seeds, DEX and ST on pain responses in formalin test. (A) In initial phase (B) and the late phase of FT, administrating the aqueous extract of *CS* (125, 250, 500 and 1000 mg/Kg IP), DEX (0.5, 1 and 2 mg/Kg IP) or ST 30 minutes before the FT test decreased the time of licking and climbing foot’s behavior (p < 0.01). Besides, the pretreatment of NAL reversed this effect. Data are expressed as the mean ± S.E.M. *p < 0.01, **p < 0.001 as compared with Cont or VEH groups, # p < 0.01 as compared with CS 1000.

The results of formalin test are shown in Figure 5 (A and B). In the formalin test, the CS extract, DEX and ST has antinociceptive effect in both phases of formalin test (p < 0.01) through decreasing the reaction time in comparison to the vehicle-treated and control animals in the early (A) and late (B) phases. In addition, animals that received 1000 mg/Kg of CS extract had the best reaction time in comparison with the other groups and the pretreatment of NAL (2 mg/Kg) at 1000 mg/Kg increased the reaction time in the early and late phases of formalin test. Thus, NAL attenuates the antinociceptive effects of the CS extract.

The main purpose of the present study was to investigate the role of opiate system in the antinociceptive effect›s of CS on acute and chronic pain in mice by HP, Tail flick TF and FT. Also compares antinociceptive effects of CS with that of DEX and ST on pain. Our results indicate that systemic injection of the aqueous extract of CS seeds can modulate the acute and chronic pain dose-dependently as indicated with the increased reaction time in tail flick and hot plate models and the decreased pain responses in both phases in formalin model. These results also showed that the analgesic effects of the CS extract were in the range of 125 to 1000 mg/Kg. But in dose of 1000 mg/Kg, CS extract was the most effective and also more potent compared to the other analgesic agents (DEX and ST). These findings demonstrate that the CS extract plays an important role in modulating the acute and chronic pain.

Furthermore, our results showed that DEX (as an agonist of glucocorticoid receptor that released the hypophyseal endorphins and junction to receptors) and ST ( which activates the hypothalamus-pituitary-adrenal axis and causes a release in glucocorticoids) have analgesic effects which confirmed the results of the previous studies (23, 24, 40, 41).

Although the modern pharmacological studies has demonstrated that antihypertensive (5), antifertility (6), antihyperglycemic (7-11), antihyperlipidemic (12, 13), antiproliferative (14), antioxidant (15, 16), anticonvulsant (17), antinociceptive (18,19) and anxiolytic (20) effects of this medicinal plant decrease the toxic heavy metal accumulation in fish (22), there is no pharmacological report about the role of opiate system in antinociceptive of CS properties.

Chemical studies on CS have shown constituents such as quercetin 3-glucoronide linalool, camphor, geranyl acetate, geraniol, and coumarins (42-44). Linalool is a monoterpene compound commonly found as a major component of the essential oils of several aromatic plant species, many of which are used in traditional medical systems as analgesic and anti-inflammatory remedies (45).

No studies on the biological fate of coriander oil per se in the human body have appeared, but in animal studies, one of the major constituents of the oil, linalool, is rapidly absorbed, metabolized and excreted from the body. Coriander oil and its major constituent, linalool, have low acute oral and dermal toxicity in laboratory animals (46).

In recent studies, it has been shown that linalool has anti-inflammatory, antihyperalgesic and antinociceptive effects in different animal models (46-49). It has been found that the administration of linalool can increase the animal reaction time in the hot plate test. In addition, it produces a significant reduction in the neurogenic and inflammatory phase of the formalin model (47). Pretreatment with atropine, a muscarinic receptor antagonist, naloxone, an opioid receptor antagonist, sulpiride, a dopamine D2-receptor antagonist, and Glibenclamide, an ATP-sensitive potassium channel inhibitor, have been shown to be able of decreasing these effects of linalool (48). Thus, it seems that the antinociceptive effects of linalool are due to its ability to stimulating the opioidergic, cholinergic and dopaminergic systems, as well as to its interaction with potassium channels or to blocking the glutamate NMDA receptors (47). Moreover, in a recent study it has been indicated that the pretreatment with 1,3-dipropyl-8-cyclopentylxanthine (DPCPX), a selective adenosine A_1_-receptor antagonist and 3, 7-dimethyl-1-propargilxanthine (DMPX), a selective adenosine A_2A_-receptor antagonist, significantly depresses the antinociceptive effects of linalool (49). This finding demonstrated that A_1_ and A_2A_ adenosine receptors are involved in linalool-induced analgesia (49).

In formalin test which have two distinct phases, the initial phase is neurogenic and the late one is inflammatory phase (38, 50). In late phase, several mediators such as histamine, kinin, serotonin and prostaglandins are released from damaged cells which take part in the inflammatory response and are able to stimulate the nociceptors and induction of pain (51). In this test, the centrally acting drugs such as narcotics inhibited both phases equally, while peripherally acting drugs only inhibited the second phase (52). The effect of the CS extract on the second phase of formalin test (inflammatory phase) indicated that CS may have anti-inflammatory effect.

Our results showed that the time spent in licking and biting of the injected paw was significantly reduced through the intraperitoneal administration of the CS extract in both phases. In fact, the effect of the extract on both phases showed that they contain active analgesic principles acting both centrally and peripherally.

On the other hand, our result showed that Naloxone reversed the antinociceptive effect of the extract in both phase in the formalin test. This finding indicated that the opioid system (at least partially) involves in the antinociceptive action.

The hot plate test is used for assessing the central antinociception activities and is a supraspinally organized response of pain (33, 53, 54) and a central model that has a selectivity for opioid-derived analgesics (55). The administration of the CS extract showed a potent antinociceptive effect. In this test, pretreatment with Naloxone reversed this antinociceptive effect confirming that the antinociceptive effect is produced through the activation of the opioid system. The central analgesic effect of the extract may be supported with the results recorded in the tail flick test which is a selective method capable of screening the centrally acting opiate analgesic drugs. This test is very sensitive to centrally acting drugs (56).

In conclusion, this study demonstrates the analgesic activity of the CS extract which is parallel as a traditional use of this extract as an analgesic and anti-inflammatory medicine. The extract (1000 mg/Kg) was more effective and more potent than DEX and ST. The mechanism of the CS seeds action is partially due to the presence of linalool as a main constitute of it and acts partly through an opiate-mediated mechanism. It seems that the opiate receptors involve in the antinociceptive effects of the CS aqueous extract.

*Declaration of interest*

The authors report no conflicts of interest. The authors alone are responsible for the content and writing of the paper.
